# Evidence-Based Analysis of Social Impact Bonds for Homelessness: A Scoping Review

**DOI:** 10.3389/fpsyg.2022.823390

**Published:** 2022-06-20

**Authors:** Huan Wang, Xiaoguang Xu

**Affiliations:** College of Economics, Shenzhen University, Shenzhen, China

**Keywords:** scoping review, social impact bonds, innovative financing mechanisms, evidence-based analysis, homelessness

## Abstract

Social impact bonds (SIBs) have emerged as an innovative financial instrument designed to support the social service sector in delivering innovative social programs. In particular, SIBs can be used to finance prevention of homelessness among those regarded as vulnerable. There is little evidence that outcomes from SIB-funded programs are significantly different compared to more traditional programs. This is the first scoping review of academic and gray literature that explores the main features and outcomes from all SIBs for homelessness based on evidence, addressing an important gap in the literature. The scoping review provides a transparent and comprehensive approach for mapping areas of this research. A total of 73 studies and articles were found eligible for inclusion. These concerned 32 SIBs for homelessness implemented in the United Kingdom, the United States, Australia, and Belgium. The review found that academic papers on SIBs for homelessness lack evidence-based analysis, while gray literature lacks transparency, especially in evaluation method and outcome. We found that fourteen projects met their target outcomes. The common features of these SIBs were a navigator intervention model, effective partnership working, and use of Special Purpose Vehicles. Our findings show that it is necessary for the managers of SIBs to improve outcome metrics and evaluation methods, support target groups fairly, and attract more private investors to finance SIBs for better addressing homelessness.

## Introduction

Homelessness has become a global problem. According to Global Homelessness Statistics, 1.6 billion people worldwide live in poor housing conditions, with about 15 million being forced to relocate every year. In the context of ongoing economic uncertainties across the world, post-COVID 19, this number is expected to grow in the absence of effective policy intervention (Aldridge and Enevoldsen, 2021).

Globally, there is no consistent definition of homelessness. Researches argue that definitions of homelessness do and should vary to offer different perspectives on homelessness based on criteria such as country, lifestyle, location, permanence of occupation, welfare entitlement, and housing quality (Tipple and Speak, 2005). The common characteristic of the definitions is that homelessness is the condition of lacking stable, safe, and adequate housing (Tipple and Speak, 2005; Toro, 2010; Wallace et al., 2018; Clifford et al., 2019, 2022; Sadzaglishvili and Kalandadze, 2019). Amore (2013) argues homelessness should be replaced by the concept of severe housing deprivation, which includes two main criteria: (1) that a person is living in severely inadequate housing due to (2) a lack of access to housing that meets a minimum adequacy standard. Amore’s definition is used in this paper, as it is a more comprehensive definition of homelessness.

Homelessness is a complex public health and social problem that is both a driver and a consequence of ill-health, social exclusion and economic marginalization (Vallesi et al., 2019). Physical ill-health is a concerning issue amongst homeless populations. They have a myriad of health problems, including high rates of chronic diseases, intentional and unintentional injury, and mental health and substance use problems (Yoshioka-Maxwell and Rice, 2019). The average life expectancy of people who experience homelessness is 30 years less than non-homeless populations. Homeless populations have high rates of mental illness (Fraser et al., 2019). Homeless people experience greater levels of discrimination and stigma, the negative effects of which make it difficult for people to escape homelessness (Mejia-Lancheros et al., 2021). In addition to human suffering, public expenditures associated with homelessness are substantial. Few rigorous studies quantify the additional social losses in productivity and well-being (Fowler et al., 2019). Countries around the world struggle to manage the human and financial burdens of homelessness. Trends in homelessness remain stubbornly high despite policy initiatives to end homelessness (Abdel-Samad et al., 2021).

In 2011, a new homeless support service navigator model was developed using Social Impact Bonds (SIBs). This endeavor involved the Department for Communities and Local Government, the Greater London Authority (GLA), homeless organizations, and others besides (Mason et al., 2017). SIBs are an innovative financing mechanism that transfers fiscal risk from governments or commissioners to new investors who provide up-front funding to expand evidence-based social programs and improve outcomes for vulnerable populations (Berndt and Wirth, 2018). According to the Impact Bond Global Database and the University of Oxford Government Outcomes Lab, several national governments, including those of the United Kingdom (UK), United States (US), Australia, and Belgium, have developed SIBs for funding the prevention of homelessness.

The SIB model offers opportunities, challenges, and obstacles under active discussion by many scholars and practitioners (Trotta et al., 2015; Rania et al., 2020; Warner, 2020) but far less headway has been made in analyzing the evidence supporting the use of SIBs for financing the prevention of homelessness. Fraser et al. (2018a) did an international review of the use of SIBs suggests that there is a paucity of concrete evidence about outcomes, where much of the reportage on SIBs is commentary and speculation. Painter and Culhane (2021) review Social Impact Bonds. They note that it is difficult to determine whether the SIBs can help accelerate public sector reform to end homelessness. However, Painter and Culhane (2021) just summarize the opinions of other researchers’ lack of evidence-based analysis.

Wang et al. (2018) reconstruct the evaluation model based on the SIB solving homeless problems in the United Kingdom by weight function. The limitation is that reconstructed evaluation model is not validated by the real cases. Vallesi et al. (2019) evaluate the Journey to Social Inclusion program in a randomized controlled trial (RCT) that aims to test the effectiveness of the program relative to standard service provision. The study is conducted in Australia and findings may not generalize to other nations (Vallesi et al., 2019). George et al. (2020) focus on the views of link workers in a SIB funded project which works with rough sleepers in the East of England. The study concludes that if SIBs are effective solutions to deeply ingrained social problems, there needs to be more careful evaluation of their true benefits in comparison to publicly funded projects. Wirth explore the functioning and implementation of a social impact bond-funded welfare service for young homeless people in the United Kingdom (Wirth, 2021). The empirical case studies for this article are only a group of youth homelessness projects called the Fair Chance Fund Social Impact Bonds. Aldridge and Enevoldsen (2021) set out the policy context on homelessness and street homelessness in England and to provide an overview of how improvements to the available data and evidence have contributed to, and influenced, policy-making. While the evaluation suggests that the SIB worked effectively, the program only took place in London (Aldridge and Enevoldsen, 2021).

In a growing critical literature on SIBs, a largely doubt whether the SIBs can help accelerate public sector reform end social problems. Existing studies about SIBs for homelessness are lack of evidence-based analysis, or narrowly focus on a single case study that the findings may not generalize to other programs in other countries. Thus, this scoping review focuses on evidence-based analysis and explores the key features and outcomes of all SIBs for homelessness. Specifically, it will address the following research objectives: (1) Analyze academic and gray literature relating to SIBs launched for homelessness. (2) Develop a unique database summarizing target groups, interventions, investment, financial terms, evaluation, and outcomes of all SIBs for homelessness. (3) Explore the key features and outcomes of SIBs issued for homelessness.

## Methodology

A scoping review was employed as the lack of high-quality research in new research field meant a systematic review was not feasible (Levac et al., 2010). Key defining features that comprised a working definition of scoping studies included the exploratory mapping of literature in a field, iterative process, inclusion of gray literature, and no quality assessment of included studies (O’Brien et al., 2016). Unlike traditional systematic reviews, scoping reviews are not intended to assess the quality of existing literature, but to provide context for a comprehensive systematic review of a research area, or to identify areas of literature where existing research is sparse (Brien et al., 2010).

We conducted a scoping review in order to synthesize the evidence of the key characteristics and outcomes of the implemented SIBs for homelessness. We used a scoping review methodology to map the SIBs for homelessness as the field is nascent, publication themes are widely scattered, and conventional searches of academic databases are less likely to be fruitful.

This scoping review is based on the checklist of Preferred Reporting Items for Systematic Reviews and Meta-Analyses Extension for Scoping Reviews (PRISIMA-ScR) (Tricco et al., 2018; Hulse et al., 2021), and on guidance for conducting systematic scoping reviews (Peters et al., 2015). The methodological framework for this scoping study contains information sources, eligibility criteria, search strategy, and critical appraisal.

### Information Sources

Arksey and O’Malley (2005) emphasize the importance of comprehensiveness in identifying relevant studies. As the study of SIBs is a nascent field and lacks academic analysis, we gather information from both academic and gray literature and complement it with knowledge from key informants. The following electronic databases were searched for this scoping review: Web of Science, Elsevier, Google Scholar, and Google.

### Eligibility Criteria

Eligibility criteria for the scoping review search are listed in Table 1. We searched for articles published from 2010 to 2022 that related to the features and outcomes of SIBs for homelessness. The start date of 2010 was chosen because that was the year the first SIBs were launched by the United Kingdom.

**TABLE 1:** Eligibility criteria.

Criterion	Inclusion	Exclusion
Time period	2010–2022	Studies outside these dates
Language	English	Studies not available in English
Academic and gray literature	Focused on SIBs for homelessness	Not related to SIBs for homelessness

To be eligible for inclusion, all academic and gray literature needed to be published in English and focused on SIBs for homelessness. The eligible types of gray literature were databases, working papers, fact sheets, reports, and webpages of related stakeholders. This review covered all SIBs for homelessness that were published through the databases of Social Finance and the University of Oxford Government Outcomes Lab. Based on those two databases, a unique database was created summarizing the target groups, interventions, investment, financial terms, evaluation, and outcomes of all SIBs for homelessness. As some information in the source databases was incomplete or not updated, it was necessary to supplement the data using other gray literature like evaluation reports or outcome reports.

### Search Strategy

The SIBs are referred to as Payment by Results (PbR) instruments in the United Kingdom, the Pay for Success (PFS) model in the United States, and Social Benefit Bonds (SBB) in Australia (Trotta et al., 2015; Edmiston and Nicholls, 2018). Keywords for the literature search were ‘social impact bond*,’ ‘payment by result*,’ ‘pay by result*,’ ‘pay for success*,’ ‘social benefit bond*,’ ‘pay for performance *,’ ‘impact investing*,’ ‘impact bond*,’ ‘homelessness impact bond*,’ and ‘homelessness*’. Both published and gray literature were identified in the search, titles, abstracts, and full text of articles were reviewed for relevancy and eligibility under the inclusion and exclusion criteria.

### Critical Appraisal

Gray literature sources are not subject to peer reviewing and do not have the same rigorous as published sources. Gray literature in this scoping review was critically appraised via the authority, accuracy, coverage, objectivity, the date, and significance (AACODS) checklist (Tyndall, 2010). AACODS checklist is designed to evaluate authority, accuracy, coverage, objectivity, the date and significance of the gray literature sources.

## Results

**FIGURE 1:**
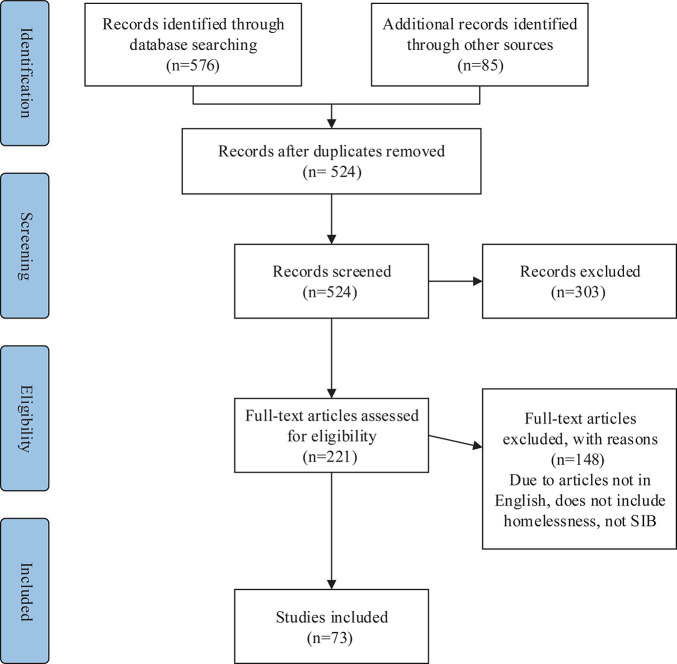
PRISMA flow diagram of screening process and outcome (Moher et al., 2009).

Figure 1 outlines the screening process applied to identified studies. A total of 576 articles were identified from Web of Science, Elsevier, and Google Scholar, and 85 articles were obtained from Google. In the end, 73 articles were included in the scoping review.

### Summary of Academic and Gray Literature

A total of 73 articles were included in the scoping review, of which 14 were academic articles and 59 gray literature. We found that the body of published work exploring evidence from SIBs launched for homelessness remains inadequate.

Regarding the 14 included academic articles, most were published in 2018 and 2019 (7/14), reflecting the emerging nature of this field (Cox, 2011; Cooper et al., 2013; Cooper et al., 2016; Carrillo, 2017; Andreu, 2018; Edmiston and Nicholls, 2018; Finn et al., 2018; Ramsay and Tan, 2018; Wang et al., 2018; Scognamiglio et al., 2019; Vallesi et al., 2019; George et al., 2020; Painter and Culhane, 2021; Wirth, 2021). The articles were authored in the United States (4/14), the United Kingdom (3/14), Australia (2/14), Italy (1/14), Ireland (1/14), Switzerland (1/14), and China (1/14). There was one cross-country study involving the United Kingdom and Canada (1/14), and one compared analysis with other financial tools (1/14). Most employed quantitative analysis (10/14), mainly focusing on single case studies and interviews. Just two articles involved qualitative analyses, and one utilized mixed methods, including both quantitative and qualitative analysis (Vallesi et al., 2019).

Regarding gray literature, all of them were critically appraised via ACCODs checklist to mitigate the risk of bias. Most were identified from online databases, namely, Social Finance and the University of Oxford’s Government Outcome Lab (32/59). Other included literature consisted of reports published by investors, commissioners, evaluators, service providers, government departments, social research firms, universities, and other non-profit organizations (18/59), or was obtained from the websites of investors, service providers, and commissioners (7/59), a fact sheet (1/59), and an unpublished thesis (1/59).

### Target Groups of Social Impact Bonds for Homelessness

As of April 2022, 32 SIBs had been issued for homelessness in four countries. Most (22) were issued in the United Kingdom. The remainders comprised five in the United States, four in Australia, and one in Belgium. The first SIB for supporting homelessness was issued in 2011 in the United Kingdom. The majority of included SIBs were issued from 2015 to 2020 (29/32; 91%). Collectively, all 32 had a total of over 23K service users, of which about 20K were in the United Kingdom, nearly 1,865 in the United States, 1,080 in Australia, and 133 in Belgium. Importantly, the Social Finance database and that of the University of Oxford’s Government Outcome Lab are not up to date. For example, the Los Angeles SIB is not included in either but instead is disclosed in one unpublished thesis (Johnson, 2019). Consequently, we needed to refer to gray literature to complement and update the database information.

The target groups of all SIBs supporting homelessness are summarized in Table 2. The various SIBs targeted people of different ages. For example, 11 SIBs (34%) targeted young people aged between 18 and 24 who were not in employment, education, or training (NEET) or were at risk of homelessness, while only two SIBs supported seniors, and just one targeted homeless individual aged 15–18 years. This finding shows that vulnerable children or seniors are not the main target groups of SIBs, particularly compared to young people aged 18–24. However, young people do not predominate among the homeless. For instance, it has been reported that in the United States, over 18% of homeless people are children, approximately 8% are between the ages of 18 and 24, and approximately 74% are over the age of 24 (Henry et al., 2021). Therefore, more SIBs should be implemented to solve the problems of vulnerable children and seniors.

**TABLE 2:** Target population and interventions of all SIBs for homelessness to date.

Country	Stage of development	Target population	Intervention
United Kingdom	(1) Completed project: ACTion Glos (Assertive Community Treatment in Gloucestershire), ACTion Lincs (Lincolnshire), Ambition (Leicestershire), Aspire (Gloucestershire), Depaul (Greenwich), Fusion (West Yorkshire), Home Group (Newcastle), Local Solutions (Liverpool), St Basil’s (Birmingham), London Homelessness Social Impact Bond (St Mungo’s), London’s SIB (Thames Reach), Greater Manchester’s SIB. (2) Ongoing project: Mayday Inspire (Northamptonshire), London’s SIB, Newcastle and Gateshead’s SIB, Single Homelessness Prevention Project (SHPS) Brent, Brighton’s SIB, Bristol’s SIB, Kirklees Integrated Support Services, Opening Doors (Bexley), Promoting Independence (Sheffield), Single Homeless Prevention Service (London).	(1) Young people not in employment, education, or training. (2) People having slept outside for long periods of time. (3) Rough sleepers with complex needs. (4) Adults at risk of being homeless. (5) Single adults and childless couples living in temporary accommodation.	(1) Housing First approach. (2) Delivery model based on a team approach. (3) Specialist link workers provide personalized support. (4) Supports a focus on sustaining accommodation, and on employment, training.
United States	(1) Completed project: Massachusetts’ SIB, Denver’s SIB. (2) Ongoing project: Los Angeles County’s SIB, Santa’s SIB.	(1) Individuals experiencing chronic homelessness. (2) Anticipated high-cost users of emergency services. (3) Individuals with histories of homelessness and involvement with the criminal justice system. (4) Single and residing in an emergency shelter.	(1) Home and Healthy for Good program. (2) Permanent supportive housing and assertive community treatment. (3) Rapid rehousing. (4) Homes Not Jail program.
Australia	Ongoing project: Aspire Social Impact Bond Adelaide, Journey to Social Inclusion (Victoria), The Youth CONNECT Social Benefit Bond (Queensland), Foyer Central SIB (Sydney)	(1) Individuals at risk of homelessness, released from a partnering prison, or discharged from a partnering hospital. (2) People experiencing sustained and chronic homelessness. (3) Young people exiting the child protection system. (4) Young people leaving out-of-home care and at risk of or experiencing homelessness.	(1) Housing First approach. (2) Trauma-informed intervention that integrates intensive case management and service coordination. (3) A relationship-based approach and provision of long-term support. (4) Connect with education, training, and employment.
Belgium	Ongoing project: Back on Track (Belgium)	(1) Young adults without income or accommodation, or released from prison.	(1) Housing First for Youth program.

In conclusion, most SIBs to date have targeted individuals who have slept outside for long periods of time or experienced multiple episodes of homelessness. As some homeless individuals have complex needs, including substance misuse issues, mental health issues, recent offending history, physical health issues, and learning difficulties, appropriate targeting and screening of participants is an important consideration.

### Interventions of Social Impact Bonds for Homelessness

Extant SIBs for homelessness in the United Kingdom, the United States, Australia, and Belgium all take the Housing First approach, which is increasingly being recognized internationally as the most effective model in helping homeless people into settled accommodation, has been proven to be a more cost-effective way of addressing homelessness than traditional model (Ly and Latimer, 2015). The Housing First approach does not require that participants meet preconditions for entry such as entering treatment, achieving sobriety, or committing to ongoing service participation requirements (Mary et al., 2018). The Housing First approach aims to quickly and successfully connect individuals experiencing homelessness with a settled accommodation. In addition, the United Kingdom, the United States, Australia, and Belgium arrange for specialist link workers to provide personalized interventions. An individual each participant can turn to specialist link workers for help with wider life issues such as budgeting, health, offending, drug and alcohol addiction, or relationships. The role of a link worker is to flexibly support clients to meet all of their needs in any setting, whether that be the street, hospital, prison, or home. Notably, interventions in the included SIBs were mainly delivered by not-for-profit organizations with specialized experience. Only the providers of Gloucestershire’s SIB and Liverpool’s SIB were private for-profit companies. It shows that not-for-profit organizations are the main service providers of SIBs for homelessness.

The projects in the United Kingdom were delivered by a team of specialists that included a drug and alcohol recovery worker and a mental health practitioner. These projects benefit from significant contributions by local housing, education, and other support organizations, enabling expertise to be joined and tailored to each vulnerable individual to obtain the best outcomes. Academic articles have also demonstrated that collaboration of several involved actors within a project is very important (Smeets, 2017; La Torre et al., 2019; Jamieson et al., 2020). Projects in the United States realized that lack of stable housing is associated with significant health concerns and used the Home and Healthy for Good (HHG) model, in which supportive housing is paired with medical and mental health services, substance abuse treatment, and vocational training, all coordinated by a case manager (Golden et al., 2015; Brown et al., 2019; Johnson, 2019). Those in Australia seek to build independence and resilience through tiered services including employment pathways, life skill development, and connection to broader services (Vallesi et al., 2019; Riddell, 2020). As of February 25, 2020, BNP Paribas listed on its website that endeavors in Belgium focus first of all on housing, then helping young people to recover, build networks, and find jobs or training.

Current literature indicates that a focus on housing is effective in addressing homelessness (Gulcur et al., 2003; Schiff and Rook, 2012; Pleace, 2018). Another article observed housing to be followed by significant reductions in client use of public services (Finn et al., 2018). In addition, the “navigator” model was rather than only pursing a “housing first” strategy. This intervention emphasized the idea that intense personalized interventions and sustained support provided by a navigator link worker should be given priority (Wirth, 2020). Navigators link workers can provide intensive, practical, and psycho-social support on the basis of individually-tailored action plans informed by completion of outcomes. Thus, it is not a traditional intervention but rather provides persistent practical and emotional support across the landscape of existing provision (Mason et al., 2017). In conclusion, although the housing first model is effective model in helping homeless people into settled accommodation, housing first model could be paired with navigator intervention model, which is effective in supporting homelessness with high levels of complex needs.

### Investment Into Social Impact Bonds for Homelessness

Table 3 summarizes the investments made into SIBs financing homelessness in the United Kingdom, the United States, Australia, and Belgium. The lowest investment was US$0.13 M into Manchester’s SIB in the United Kingdom, and the highest was US$10 M into Los Angeles County’s SIB in the United States (Lantz and Iovan, 2018). The average initial investment was US$2.29 M. Five funds were listed as financing 19 SIBs: the Fair Chance Fund (7/19), Rough Sleeping Program (5/19), Life Chances Fund (4/19), GLA Rough Sleeping Program (2/19), and Commissioning Better Outcomes Fund and Social Outcomes Fund (1/19).

**TABLE 3:** Investment in SIBs for homelessness.

Country	Capital raised	Fund	Investor types
United Kingdom	(1) Total amount: over US$23M. (2) Lowest amount: US$0.13M for Manchester’s SIB. (3) Highest amount: US$4.432M for Kirklees Integrated Support Services.	Fair Chance Fund, Rough Sleeping Program, Life Chances Fund, GLA Rough Sleeping Program, and Commissioning Better Outcomes Fund and Social Outcomes Fund.	(1) Charity/non-profit organizations (16/32). (2) Social merchant banks (12/32).
United States	(1) Total amount: over US$23M. (2) Lowest amount: US$3.5M for Massachusetts’ SIB. (3) Highest amount: US$10M for Los Angeles County’s SIB.	Undisclosed	(3) Impact investment companies (11/32). (4) Private investors (10/32). (5) Philanthropic foundations (8/32).
Australia	(1) Total amount: over US$18M. (2) Lowest amount: US$ 3.83M for Queensland’s SIB. (3) Highest amount: US$9M for Aspire’s SIB.	Undisclosed	(6) Private foundations (2/32). (7) Commercial banks (2/32). (8) Insurance companies (2/32).
Belgium	Total amount: $1.90M	Undisclosed	

As listed in Table 3, the most common investor type was charity/non-profit organizations (16/32), followed by high-worth social merchant banks (12/32), impact investment companies (11/32), private investors (10/32), philanthropic foundations (8/32), private foundations (2/32), commercial banks (2/32), and insurance companies (2/32). Investors can be divided into two categories, senior and subordinate investors, with senior investors being able to get higher interest rates than subordinate investors. For example, the Santa Clara County project is expected to return 5% interest to senior investors and 2% interest to subordinate investors if outcome metrics are met (Johnson, 2019). Some SIB service providers are also investors, such as the P3 Charity (People Potential Possibilities), which participated in Ambition (Leicestershire), Aspire (Gloucestershire), and the London Homelessness Social Impact Bond (St Mungo’s and Thames Reach). Some invested into more than one SIB. For instance, the organization Big Issue Invest invested into Ambition, Depaul (Greenwich), Local Solutions, St Basil’s, the London Homelessness Social Impact Bond (St Mungo’s and Thames Reach), the Entrenched Rough Sleepers Social Impact Bond- Pan-London, the Entrenched Rough Sleeping Social Impact Bond- Newcastle and Gateshead, the Entrenched Rough Sleepers Social Impact Bond- Street Impact Brighton, Opening Doors (Bexley), and Promoting Independence (Sheffield).

We could not find data on the investors of ACTion Glos, ACTion Lincs, Journey to Social Inclusion, or the Youth Connect Social Benefit Bond (Queensland). In addition, information on the investors of some projects published by Social Finance is incomplete and needs supplementation by data from the Government Outcomes Lab. Those projects were Ambition, Aspire, Depaul, Home Group, and the London Homelessness Social Impact Bond (St Mungo’s). Moreover, some information in the two source databases is inconsistent. For example, Salt Lake County’s investment as disclosed in the Government Outcome Lab is US$5.5M, while that disclosed in Social Finance is US$4.4M. Bristol’s investors were also inconsistently reported in the two databases. Therefore, key informants should be contacted to identify an investment’s corresponding investors.

### Financial Terms of Social Impact Bonds for Homelessness

Table 4 provides an overview of the outcome metrics of all SIBs for homelessness. All used at least one target outcome. Metrics tied to payment included outcomes based on accommodation, wellbeing, education/training, employment, independence, or days in jail (Brown et al., 2019). Outcome payers were mainly local or central governments (31/32). However, the outcome payer of Journey to Social Inclusion (Victoria) was non-governmental (1/32). SIBs are important for allowing local governments to embark on innovative homelessness projects while minimizing financial risk and limiting resource commitment by the federal government. Notably, one report and thesis indicated that some tracked outcomes are not tied to success payments (Brown et al., 2019; Johnson, 2019). For instance, in the Massachusetts SIB, health care service usage, number of nights spent in shelter, and number of days incarcerated were tracked but not tied to any success payment. Taking this implementation as a model will allow future SIBs to adjust outcome metrics as appropriate.

**TABLE 4:** Financial terms of all SIBs for homelessness.

Country	Outcome metrics	Maximum return	Interest rate	Structure
United Kingdom	(1) Sustained accommodation. (2) Sustained reconnection. (3) Improved health and wellbeing. (4) Improved education/training. (5) Achieved independence. (6) Reduced rough sleeping.	Gloucestershire: $1.4M, Lincolnshire: $1.8M, Leicestershire: $4.1M, ACtion Glos: $2.1M, Greenwich: $2.2M, Liverpool: $1.7M, Birmingham: $3.4M, London’s SIB (St Mungo’s and Thames Reach): $1.6M, Newcastle and Gateshead: $2.1M, Bristol: $6.8M, Bexley: $2.3M, Brent: $1.6M, and Newcastle: $3.2M. Other projects undisclosed.	(1) ACtion Glos: 20% discount to the maximum outcome payment rate-card. (2) London’s SIB (St Mungo’s and Thames Reach): annual rate 6%. (3) Other projects undisclosed.	(1) Intermediated structure: Leicestershire, Gloucestershire, Greenwich, West Yorkshire, Newcastle, Liverpool, Birmingham, East and South East London, West and North West London, Manchester, and Single Homeless Prevention Service. (2) Other projects have directed or undisclosed structure.
United States	(1) Stable tenancy. (2) Decreased jail bed days. (3) Reduced rate of re-incarceration. (4) Enrollment into substance abuse service. (5) Accepted mental health services.	Massachusetts: $6M, Santa Clara: $12, Denver: $11.4M, Los Angeles: $11.5, Salt Lake County: $5.5M.	(1) Massachusetts: maximum return of 5.3%. (2) Santa: senior investor 5%, subordinate investor 2%. (3) Denver: 3.5%. (4) Los Angeles and Salt Lake County: senior investor 5%, subordinate investor 2%.	(1) Intermediated structure: Santa Clara County, Denver, Los Angeles County, Salt Lake County, and Massachusetts.
Australia	(1) Hospital bed days. (2) Convictions. (3) Crisis accommodation periods. (4) Stable housing, employment, or education. (5) Improved health and wellbeing. (6) Personal development.	Aspire: $12M, Foyer Central SIB: maximum return 10%. Victoria and Queensland: undisclosed maximum return.	(1) Aspire: below target 4.5%, target 8.5%, above target 12%, fixed coupon rate is 2% per annum. (2) Foyer Central SIB: below target 1.0%, target 5.9%, above target 9.6%, and fixed coupon of 2% per annum for first 3 years. (3) Victoria and Queensland undisclosed.	(1) Intermediated structure: Adelaide, Victoria, and Queensland. (2) Directed structure or undisclosed: Foyer Central SIB
Belgium	(1) Obtain a renting agreement. (2) Have legal income or start training. (3) Reduction in recidivism compared to a reference rate.	Undisclosed	Undisclosed	Undisclosed

Also summarized in Table 4 are the maximum outcome payments, interest rates, and SIB structures. Projects in the United Kingdom, United States, and Australia all published return or interest rates for meeting targets. However, only Aspire and Foyer published information concerning the return to investors when outcomes are below or above the target level. If outcomes are above targets, investors can get higher returns. Besides that, an implementation agreement may be terminated early if performance is well below the target (Riddell, 2020). Therefore, it shows that outcomes meeting or exceeding the target level are important for return on investment and continuous implementation of projects.

In terms of structure, most of the included SIBs were intermediated (23/32; 72%), meaning the service provider contracted with intermediaries, particularly a Special Purpose Vehicle (SPV) like the Street Impact Project of the London Homelessness Social Impact Bond (St Mungo’s). Intermediaries can be classified as either main or secondary. For instance, in the Denver project, the main intermediary is CSH while the secondary intermediaries are Social Impact Solutions, Inc. and Enterprise Community Partners. Directed structures were also relatively common, in which the service provider contracted with the outcome payer, and some SIB structures were undisclosed (9/32; 28%). It has been suggested in an academic article that the absence of a SPV in a SIB has a negative impact on performance management when compared to SIBs having intermediate structures (Edmiston and Nicholls, 2018). This conclusion is supported by the Thames Reach SIB, which is without a SPV, and St Mungo’s Broadway, which has an intermediated structure. The majority of intermediaries were non-profit organizations (10/23). Other intermediaries included private organization (13/23), banks (5/13), private market investors (4/13), law firms (1/13), consultancy companies (1/13), and market research companies (1/13). However, intermediates were not disclosed by Gloucestershire, Lincolnshire, London, Newcastle and Gateshead, Bristol, Bexley, Sheffield, Los Angeles, or Sydney.

### Evaluation and Outcomes of Social Impact Bonds for Homelessness

According to one published article, the main evaluation methods used for SIBs are validated administrative data, historical comparisons, quasi-experimental methods, and randomized controlled trials (Gustafsson-Wright et al., 2015). As given in Table 5, the SIBs for homelessness reviewed in this study employed heterogeneous evaluation methodologies: qualitative interviews, validated administrative data, randomized controlled trials, mixed-methods approaches combining qualitative and quantitative data, before and after comparisons, and quasi-experimental approaches using propensity score matching (Cooper et al., 2013; Mason et al., 2017; Finn et al., 2018; Vallesi et al., 2019; Rosenbach and Carter, 2020; Gillespie et al., 2021).

**TABLE 5:** Evaluation methods and outcomes of all SIBs for homelessness.

Country	Evaluation method	Evaluator	Outcomes
United Kingdom	(1) Fair Chance Fund Projects (Ambition, Aspire, Depul, Fusion, Home Group, Local Solutions, St Basil’s): mixed methods approach combining the collection and analysis of qualitative and quantitative data. (2) London’s SIB (St Mungo’s and Thames Reach): qualitative evaluation and impact evaluation. (3) Manchester: the PTS approach and FCF outcomes. (4) Single Homelessness Prevention Project: interviews, surveys, and collection of management information. (5) Kirklees, Bexley, Sheffield and SHPS: semi-structured interviews. (6) Other projects undisclosed.	(1) ACTion Glos: Sheffield Hallam and Southampton universities. (2) London’s SIB (St Mungo’s and Thames Reach): International Coaching Federation (ICF). (3) Kirklees: Bridges Outcome Partnerships. (4) Bexley Sheffield and SHPS: the Government Outcomes Lab (GO Lab).	(1) ACTion Glos and Action Lincs, respectively, recruited 124 and 135 people in 2017. Sustained accommodation, mental health, and drug/alcohol support exceeded targets. (2) Fair Chance Fund Projects: accommodation, employment, volunteering, and education outcomes surpassed targets. (3) St Mungo’s and Thames Reach: stable accommodation and 13/26-week employment outcomes above target levels, reduction in rough sleeping, volunteering/employment qualifications, and reconnections with home countries lower than targeted. (4) Mayday Inspire: in the first year, over 50 rough sleepers into secure accommodation – over 30% more than initial targets. (5) Greater Manchester’s SIB: sleeping rough declined by 57% since 2017. Almost 356 people supported into accommodation. 133 people accessed mental health. 97 people accessed drug/alcohol services. 45 people started employment/volunteering. 40 people improved education/training. (6) Bristol’s SIB: at project started in 2017, 64% of 125 SIB clients were sleeping rough. Now in 2021, only 4% are on the streets.
United States	(1) Massachusetts: before and after comparison, and validated data. (2) Santa Clara County and Denver: validated data and a randomized controlled trial (RCT). (3) Los Angeles County. (4) Salt Lake County: treatment and control groups.	(1) Massachusetts: Root Cause. (2) Santa Clara County: University of California-San Francisco. (3) Denver: Urban Institute. (4) Los Angeles County: RAND Corporation. (5) Salt Lake County: University of Utah Criminal Justice Center.	(1) Massachusetts: reduction of chronic individual homelessness has significantly exceeded targets and successfully placed over 656 high-need individuals into stable, supportive housing, with 92% remaining housed after 1 year. (2) Santa housed 111 chronically unsheltered people. Annual emergency services use dropped from $62,473 to $19,767. (3) Denver: as of July 2018, two and a half years into the SIB, 85% 285 participants had remained in housing without ever exiting the program. During their first year in housing, 44 percent of participants did not return to jail. (4) Los Angeles County and Salt Lake County: undisclosed.
Australia	(1) Aspire: compares targeted and actual intervention groups. (2) Journey to Social Inclusion: mixed methods, RCT. (3) Other projects undisclosed.	Undisclosed	(1) Aspire generated total SA Government savings of $5.69 million over 3 years (to June 30, 2020), which is 210% of the initial plan. (2) Other projects are undisclosed.
Belgium	Undisclosed	A research team from KU Leuven.	Undisclosed

Eighteen projects did not disclose their evaluation methodologies. Additionally, such initiatives feature an independent evaluator tasked with assessing and reporting on performance outcomes. Among the reviewed studies, these evaluators were of many types: non-profit organization (4/32), university-based evaluators (4/32), research centers launched by universities or governments (3/32), and private or independent corporations (2/32). However, 20 SIBs did not disclose their evaluators (Mason et al., 2017; Finn et al., 2018; Johnson, 2019). Only Fair Chance Fund, London (St Mungo’s and Thames Reach), Denver, Kirklees Integrated Support Services, and Aspire Social Impact Bond Adelaide disclosed their evaluation reports.

Outcomes of the SIBs for homelessness were mainly released in the Social Finance database and through the University of Oxford’s Government Outcomes Lab. We supplemented these sources with stakeholder websites, reports, media releases, and papers. For example, commissioners of the Ministry of Housing, Communities and Local Government published an evaluation report on the Fair Chance Fund, which financed projects of Ambition, Aspire, Depul, Fusion, Home Group, Local Solutions, and St Basil’s. Likewise, the service provider Colorado Coalition for the Homeless released information about the Denver program on their website. However, just 12 projects published performance information, and some did not publish the data in full. For example, Mayday Inspire only released performance for the first year. All told, this analysis of evaluation measures and outcomes reveals that SIB projects lack transparency.

Among the 32 reviewed SIBs, 14 (44%) completed the projects. These comprised 12 projects in the United Kingdom (ACTion Glos, ACTion Lincs, Ambition, Aspire, Depul, Fusion, Home Group, Local Solutions, St Basil’s, the London Homelessness Social Impact Bond, and the Entrenched Rough Sleeping Social Impact Bond-Greater Manchester) and two projects in the United States (Massachusetts and Denver). According to the University of Oxford’s Government Outcomes Lab, only ten of these SIBs were complete, which is not accurate. Considering both completed and incomplete projects, most reported strong outcomes on accommodation, which provides evidence of the effectiveness of the Housing First model (Andrikopoulos, 2020). Employment proved to be a more popular pathway for all participants than entry into education or training. Moreover, volunteering was not a popular option in the Fair Chance Fund projects. However, volunteering was a success in terms of outcome, with positive outcomes such as improved self-esteem and reduced social isolation being reported. Overall, there is considerable evidence that SIBs improve the physical and mental health of target groups, but these aspects were not typically tracked and included in outcome metrics. The Aspire project did track the use of public services and reported a significant reduction relative to baseline in accessing justice services, emergency accommodation, and hospital bed days (Sainty, 2020). Likewise, the Santa Clara County projects housed 111 chronically unsheltered people and observed utilization of emergency services to drop by nearly $43,000 a year.

Those SIBs that met target outcomes shared several features in common: a navigator intervention model, partnership working, and use of a Special Purpose Vehicle (SPV). Evidence from project evaluations suggests that the navigator intervention model provides intense personalized services and sustained support, which is effective in supporting entrenched rough sleepers with high levels of complex needs (Mason et al., 2017). Partnership working is likewise important to meeting complex needs. For example, relationship building and partnership working with landlords and local authorities proved critical for widening access to housing options. Prior work has also reported that collaboration of several involved actors within a project is very important (Smeets, 2017; La Torre et al., 2019; Jamieson et al., 2020). Intermediary organizations that help match providers with investors, structure the financial deal, and monitor programs use a Special Purpose Vehicle. Absence of such a vehicle has been demonstrated to negatively impact performance management (Edmiston and Nicholls, 2018). Finally, the reviewed projects lack standardized reporting for describing their features and outcomes. In addition, some project-related publications were not written in English, which generated missing information. It is important for SIBs to utilize standardized reporting, thereby enabling ready comparison of their features and outcomes.

## Discussion

In a growing critical literature on SIBs, a largely doubt whether the SIBs can help accelerate public sector reform end social problems. Currently, there is little published work that explores the evidence obtained from all SIBs for homelessness. We explored that evidence utilizing both academic and gray literature and developed a unique database summarizing target groups, interventions, investment, financial terms, evaluation, and outcomes of all the SIBs for homelessness in the United Kingdom, the United States, Australia, and Belgium. This paper identified several common features of SIB studies. First, academic researches focus on the targeted outcomes, which are measurable pre-defined social outcomes and trigger payment for the SIBs, but tend to ignore soft outcomes achieved in the SIBs programs, which are difficult to measure and depend on subjective measurement, such as an individual’s self-assessment. Second, some outcomes that are tracked are not tied to success payments. Third, SIBs tend to support young people instead of the relatively more vulnerable child and senior populations. Fourth, non-profit organizations invest much more than private investors. Fifth, not all SIB-related data were disclosed. Both SIBs projects and earlier studies lack transparency.

Fraser et al. (2018b) found little evidence of SIB-funded programs having significantly different outcomes from more traditional programs (Fraser et al., 2018b). One reason for this observation is that academic studies tend to ignore soft outcomes, which are not tied to success payment but have been identified as important for supporting target population. SIBs are able to improve some soft outcomes that traditional programs cannot. For example, mental health is at the forefront of everything Fair Chance Fund projects do. In addition to the official ‘targeted outcomes’, their target population achieved a number of soft outcomes such as increased resilience, better communication skills, and improved confidence and self-esteem. While these soft outcomes were neither measured nor included in the outcome metrics, their realization should have been recognized in some way through the Payment by Result (PbR) framework. In fact, achievement of such soft outcomes has been identified as key for the achievement of education and sustained accommodation and employment outcomes, according to evaluation of the Fair Chance Fund final report 2019. Therefore, we should improve outcome metrics and evaluation methods to encompass soft outcomes, such as through conducting a qualitative study using semi-structured interviews. However, even when outcomes are tracked, they may not necessarily be tied to success payments. For instance, the Santa Clara County project tracked utilization of health care, social service, and criminal-justice systems, but did not tie these measures to payments even though health conditions and crime reduction are also important for addressing homelessness. In the future, the current array of inadequate and imperfect outcome metrics needs to be improved. Moreover, the problem of imperfect metrics could be addressed by utilizing the payment mechanism divided between a fixed and a variable payment per project (Andersen et al., 2020).

Our review revealed that SIBs tend to support young people aged 18–24, instead of the more vulnerable child or senior populations. This is not consistent with age demographics among the homeless. For example, a prior study in the United States reported that over 18% of homeless people are children, approximately 8% are between the ages of 18 and 24, and approximately 74% are over the age of 24 (Henry et al., 2021). One reason for preferentially supporting young people is that doing so can yield better performance in the short term. There is a risk of “cream-skimming” if providers offer services only to those who are most likely to benefit from intervention (Cox, 2011). In addition, non-profit organizations that act as service providers aim to address inequities and help vulnerable groups fairly. As such, primarily supporting young people goes against the purpose of these organizations. Overall, SIBs are morally permissible in principle but are at great risk of becoming unethical or unfair in practice (Morley, 2021). Going forward, more SIBs are needed to support vulnerable children and seniors rather than tending to support young people.

It is noteworthy that among the 32 SIBs reviewed here, non-profit organizations comprised more of the investors (16/32) than did private investors (10/32). This finding is consistent with a prior academic article that reported early entrants to this new investment market as likely to be social investors for whom economic return may be less critical than social benefits (Finn et al., 2018). It is also in contradiction with some papers that regard private investors as the main investors of SIBs (Maier et al., 2018). Indeed, each SIB for homelessness featured non-profit organizations as providers, who can finance SIBs and then become investors. This may be one reason that the number of non-profit organizations as investors is higher. Another reason is that it is difficult for SIBs to attract private investors because the investment is high-risk (Giacomantonio, 2017). To promote balance among stakeholders in the SIB model, future SIBs should appeal more to private investors for project financing, such as through substantial incentives or guarantees being provided by the government or third party.

In the future, managers of SIBs should pay attention to some aspects in which SIBs still need to improve in practice. First, SIBs should improve outcome metrics and evaluation methods to capture soft outcomes, and the breadth of tracked outcomes should be tied to payments. Second, SIBs for homelessness need to support vulnerable children and seniors fairly, proportionate to the composition of the homeless population, rather than tending to support young people. Third, SIBs should increase their appeal to private investors to ensure a balance of stakeholders, such as through substantial incentives or guarantees being provided by the government or third party. Fourth, managers should publish the progress and outcome of SIBs in time so that the stakeholders and citizens can learn more about SIBs and increase the confidence of SIBs. As for the government, they need to improve the information disclosure system of SIBs, such as issuing a related decree.

The study has some limitations. First, this paper is a scoping review, which are lack of assessing the quality of existing literature, and is not as rigorous as systematic reviews, but more distinctive in methodology and is less likely to be strongly influenced by opinion than a traditional literature review. Second, some project-related publications were not written in English, which generated missing information. Due to the absence of published data, SIB outcomes were largely obtained from gray literature sources which do not have the same rigorous as published sources. Gray literature sources were critically appraised via ACCODs checklist to mitigate the risk of bias. Third, some SIBs had limited transparency in relation to some features and outcomes, which could affect the validity of the results. Therefore, some features not analyzed in this study could also have contributed to the outcomes of SIBs.

## Conclusion

This scoping review addresses an important gap in the literature that we explored the evidence provided by all extant SIBs for homelessness and analyzed their key features and outcomes based on academic and gray literature. We found that academic papers lack evidence-based analysis while gray literature lacks transparency, especially regarding evaluation methods and outcomes. All SIBs for homelessness shared several common features: a Housing First model, personalized intervention, specialized service providers, and varied investors. Fourteen of the reviewed SIBs met target outcomes. The common features of these were: a navigator intervention model, effective partnership working, and use of a Special Purpose Vehicle. These features are important for the following reasons: First, a navigator intervention model provides personalized services and sustained support for target groups. Second, having a team of specialists cooperate enables holistic support for the target population. Third, the use of Special Purpose Vehicle as the prime contractor allows supervision of service providers and isolation of financial risk.

## Author Contributions

HW collected the data and wrote the manuscript. XX was responsible for modifying the manuscript. Both authors contributed to the article and approved the submitted version.

## Conflict of Interest

The authors declare that the research was conducted in the absence of any commercial or financial relationships that could be construed as a potential conflict of interest.

## Publisher’s Note

All claims expressed in this article are solely those of the authors and do not necessarily represent those of their affiliated organizations, or those of the publisher, the editors and the reviewers. Any product that may be evaluated in this article, or claim that may be made by its manufacturer, is not guaranteed or endorsed by the publisher.
